# Performance Enhancement of a Quartz Tuning Fork Sensor Using a Cellulose Nanocrystal-Reinforced Nanoporous Polymer Fiber

**DOI:** 10.3390/s20020437

**Published:** 2020-01-13

**Authors:** Wuseok Kim, Eunjin Park, Sangmin Jeon

**Affiliations:** Department of Chemical Engineering, Pohang University of Science and Technology (POSTECH), 77 Cheongam-Ro, Pohang 37673, Korea; lucky9271@postech.ac.kr (W.K.); eunjinn@postech.ac.kr (E.P.)

**Keywords:** quartz tuning fork (QTF), electrospinning, cellulose nanocrystal (CNC), gas sensor

## Abstract

A cellulose nanocrystal (CNC)-reinforced polymethylmethacrylate (PMMA) fiber was obtained via electrospinning, and then attached between the two tines of a quartz tuning fork (QTF). The change in the resonance frequency of the CNC/PMMA composite fiber-coated QTF (CP-QTF) was measured upon being exposed to various concentrations of ethanol vapor. The frequency decreased as the ethanol vapor concentration increased, because the modulus of the composite fiber decreased due to the adsorption of the ethanol vapor. The composite fiber obtained at a high relative humidity (RH; 60% RH, CP60 fiber) produced a highly porous structure as a result of the moisture adsorption-induced phase separation of PMMA. The porosity of the CP60 fiber was higher than that of a CNC/PMMA composite fiber obtained at 30% RH (CP30 fiber) or that of a plain PMMA fiber obtained at 60% RH (P60 fiber), because hygroscopic CNCs promote moisture adsorption. The CP60 fiber-coated QTF (CP60-QTF) exhibited a greater frequency change and faster response time than P60-QTF and CP30-QTF upon exposure to ethanol vapor at the same concentration. The enhanced performance of CP60-QTF was attributed to its higher surface area and larger fiber modulus.

## 1. Introduction

Polymer-based gas sensors have attracted considerable attention as a result of their many advantages over commercially available metal oxide gas sensors, such as room temperature operation, numerous types of polymers available for sensing materials, and their low manufacturing cost [1,2,3,4,5,6,7]. Despite these advantages, they are not widely applied, because of the serious drawback that most polymers are not conductive. In order for nonconductive polymers to be utilized as gas-sensing materials, gas adsorption-induced changes in the chemical or physical properties of nonconductive polymers must be converted into electrical signals in order to enable monitoring.

A microfabricated quartz tuning fork (QTF) is a good platform to use for a gas sensor, as the intended results can be achieved by simply coating a polymer onto the QTF. Gas adsorption on the polymer affects the resonance frequency, which can be measured electrically [8,9,10,11,12]. Although its mass sensitivity (50 ng/Hz) is not as good as other competing microresonators, such as microcantilevers, surface acoustic wave sensors, and quartz crystal microbalances [13], it is cheap (US$ 0.1) and the unique geometry of a QTF, comprised of two vibrating tines with a sub-millimeter gap, allows for sensitivity enhancement by attaching a freestanding polymer nanostructure between the two tines, as small amounts of gas adsorption substantially affect the polymer modulus [14].

The sensitivity of a polymer fiber-coated QTF can be improved by increasing the surface area or modulus of the polymer fiber [15,16,17,18]. The surface area is generally increased by creating porous structures or uneven surfaces on the fiber. Of these two approaches, porous structures are preferred over uneven surfaces, because a polymer fiber with a porous structure has a greater surface area than an uneven surface, and the resulting pore network can facilitate the diffusion of gas molecules inside the polymer fiber. The sensitivity can also be improved by coating a polymer fiber with a higher modulus onto QTFs, because the gas adsorption-induced change in resonance frequency increases as the modulus of the polymer fiber increases. However, it is not straightforward to increase the surface area and modulus of a polymer fiber simultaneously, because the modulus of a polymer fiber generally decreases with increasing porosity.

We have addressed this challenge by incorporating cellulose nanocrystals (CNCs) in polymer fibers to increase both the modulus and the surface area. CNCs are crystalline materials that are typically 200–400 nm in length and 10 nm in diameter. Because of the high crystallinity, high aspect ratio, and strong hydrogen bonding between CNCs, its modulus is higher than 140 GPa [19,20,21]. In this study, we used an electrospinning method to prepare CNC-reinforced polymethylmethacrylate (PMMA) fibers. The evaporation of a volatile organic solvent during electrospinning cools the polymer fiber, which subsequently condenses the surrounding moisture into water droplets. The non-solvent (water) induces the phase separation of the PMMA solution and the resulting polymer fiber produces a nanoporous structure [22,23]. The presence of hygroscopic CNCs increases the water condensation and creates composite fibers with a higher porosity. When the CNC-reinforced PMMA (CNC/PMMA) fiber-coated QTF was exposed to ethanol vapor, the resonance frequency change was higher than the plain PMMA fiber-coated QTF, because of the increased surface area and modulus.

## 2. Materials and Methods

### 2.1. Materials

CNCs measuring ~10 nm wide and ~200 nm long were obtained from the Forest Product Laboratory, U.S. Forest Services (Madison, WI, USA). Polymethylmethacrylate (PMMA; Mw = 350,000 g mol^−1^), N,N-dimethylformamide (DMF), and dichloromethane (DCM) were purchased from Sigma Aldrich (Saint Louis, MO, USA). QTFs with a spring constant of 13 kN m^−1^ and a resonance frequency of 32.76 kHz were purchased from Sunny Inc. (Chungju, Korea). The thickness, width, and length of the QTF were 600, 300, and 3400 μm, respectively. Deionized water (18.3 MΩ cm) was obtained using a reverse osmosis water system (Human Corporation, Seoul, Korea).

### 2.2. Electrospinning of the PMMA and CNC/PMMA Fibers Under a Different RH

Two different solutions were prepared for the electrospinning of PMMA and CNC/PMMA nanofibers. For the electrospinning of the CNC/PMMA nanofibers, the water in the aqueous CNC solution was exchanged with DMF to obtain a 7.8 wt% CNC solution in DMF. Then, 0.25 g of the CNC solution in DMF, 0.65 g of PMMA, and 0.71 g of DMF were added to 5.29 g of DCM. The resulting mass ratio of CNC to PMMA was 3:100. For the electrospinning of the PMMA nanofibers, PMMA was dissolved in DMF/DCM (1:4 *v*/*v*). The PMMA concentration was determined as 12 wt%, which produced PMMA fibers with similar diameters to the CNC/PMMA fibers. After loading the solution into a 5-mL plastic syringe, electrospinning was conducted under an applied voltage of 6–10 kV and a solution flow rate of 25 μL min^−1^. The electrospun fibers were collected on two aluminum foils, which were separated by 2 cm so as to obtain freestanding fibers. The distance between the nozzle and aluminum collector was kept at 15 cm, and the relative humidity (RH) was controlled using a humidifier to maintain a 30% or 60% atmosphere [16]. The following four types of polymer fibers were produced: PMMA fibers obtained at 30% RH (P30) and 60% RH (P60), and CNC-reinforced PMMA fibers obtained at 30% RH (CP30) and 60% RH (CP60). Unless otherwise noted, the CNC content was 3 wt% of PMMA. After drying the fibers overnight in an oven at 70 °C to remove the residual solvents, the fibers were transferred onto a QTF. The fiber was glued on the QTF by dropping small amounts of acetone onto the prongs and the QTF was dried in a vacuum oven at 70 °C to remove the residual stress in the fiber.

### 2.3. QTF Measurements

A home-made LabVIEW program and a PCI-6251 data acquisition board (National Instruments, Austin, TX) were used to monitor the changes in the resonance frequencies of the QTFs upon their exposure to ethanol or water vapor. A solvent vapor stream was produced by passing dry nitrogen through a gas bubbler that contained either ethanol or water. To adjust the concentration of the vapor, the solvent vapor stream was mixed with dry nitrogen and injected into a flow cell. The flow rate of each stream was controlled using mass flow controllers (Brooks Instrument, Hatfield, PA, USA), and the total flow rate was fixed at 100 mL min^−1^. All of the experiments were conducted at room temperature.

## 3. Results and Discussion

### 3.1. Characterization of Electrospun PMMA and CNC/PMMA Fibers

**Figure 1:**
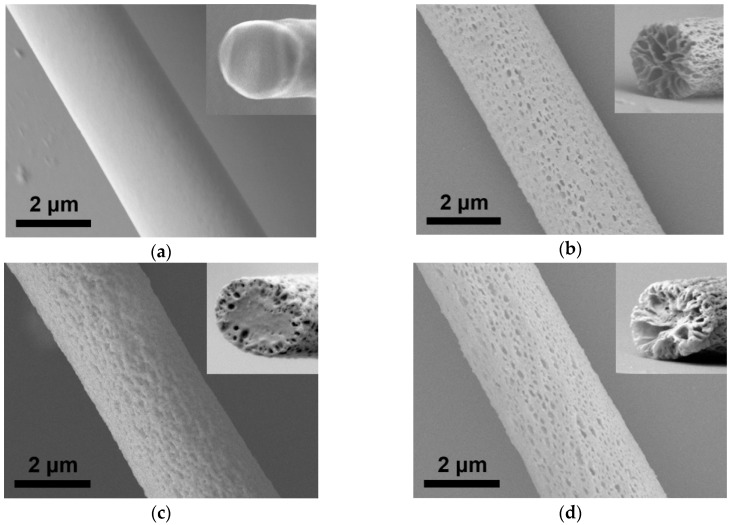
SEM images of (**a**) P30 fiber, (**b**) P60 fiber, (**c**) CP30 fiber, and (**d**) CP60 fiber. Insets show the cross-sectional SEM images of each fiber.

Figure 1a,c show the scanning electron microscopy (SEM) images of the P30 and CP30 fibers, respectively, obtained at a low humidity (30% RH). The mean diameters of the P30 and CP30 fibers were 2.71 ± 0.23 µm and 2.79 ± 0.39 µm, respectively. The P30 fibers exhibited a smooth surface, whereas surface pores were observed on the CP30 fibers. The pore size ranged from tens of nanometers to hundreds of nanometers. The porous structures were formed as a result of the presence of hygroscopic CNCs and the non-solvent (water)-induced phase separation during electrospinning. Because DCM has a higher volatility than DMF, DCM evaporates before DMF and cools the PMMA fiber during electrospinning, resulting in the condensation of moisture into water droplets. The presence of hygroscopic CNCs facilitates water adsorption. The adsorbed water mixes with DMF, but it is a poor solvent for PMMA and leads to phase separation. The cross-sectional SEM image of CP30 fibers confirmed that the pores existed only on the surface and did not penetrate the fiber completely (inset of Figure 1c). Figure 1b,d show the SEM images of P60 and CP60 fibers, respectively, which were prepared at a high humidity (60% RH). The mean diameters of the P60 and CP60 fibers were 2.71 ± 0.45 µm and 2.75 ± 0.48 µm, respectively (see Figure S1). While only surface pores were observed for the CP30 fiber, both surface pores and inner cavities were observed for the P60 and CP60 fibers because of the presence of sufficient water molecules under a high RH.

**Figure 2:**
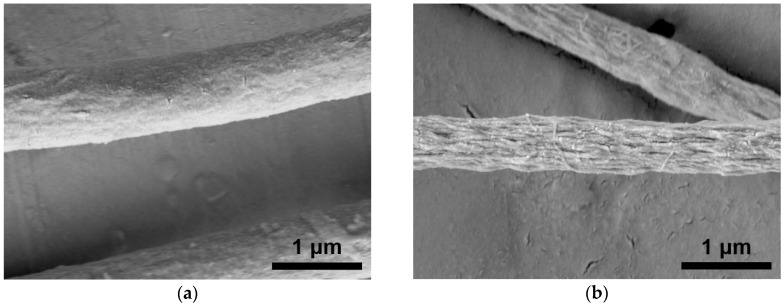
SEM images of the cellulose nanocrystal (CNC)-reinforced polymethylmethacrylate (PMMA) fibers (**a**) before and (**b**) after etching PMMA.

Figure 2a,b show the SEM images of the CNC-reinforced PMMA fiber (CNC:PMMA = 3:7 *wt*/*wt*) before and after etching the PMMA with THF, respectively. The CNCs were evenly dispersed in the fiber and aligned along the fiber axis. The high degree of CNC alignment along the fiber was attributed to the following three factors: a confinement effect, a high electrostatic field effect, and a drawing effect [24,25,26,27]. The large aspect ratio of CNCs and only a few micrometers diameter of the electrospun fiber enforced the alignment of CNCs along the fiber axis (the confinement effect). The high electrostatic force and shear force in the electrospinning process facilitated the CNC alignment along the flow direction.

### 3.2. Effect of CNCs on Reinforcement of the PMMA Fibers

**Figure 3:**
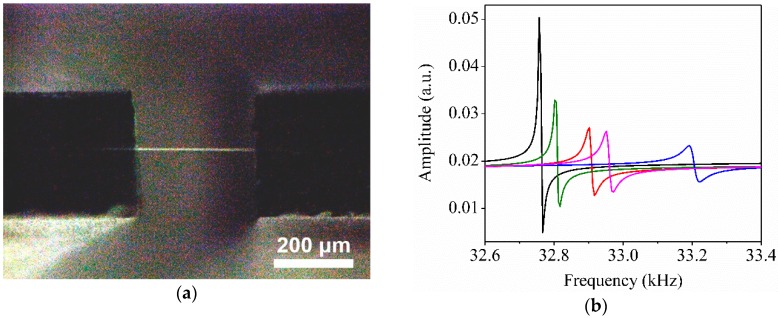
(**a**) Optical microscope image of the CP30-quartz tuning fork (QTF). (**b**) Variations in the resonance frequency peaks after mounting an electrospun fiber (black, bare QTF; red, P30-QTF; green, P60-QTF; blue, CP30-QTF; and pink, CP60-QTF).

The addition of fillers to polymers affects the mechanical properties of the composite fiber because of the reinforcing effect [28,29,30]. In particular, the addition of CNCs substantially increases the tensile modulus of the composite fiber, because crystalline CNCs have a high aspect ratio and a high modulus. Figure 3a shows an optical microscope image of the QTF coated with a P30 fiber. The resonance frequency change (Δ*f*) of a QTF is affected by the mass loading (Δ*m*) and the effective stiffness (Δ*k*) [31].

(1) $$\Delta f=\left(\frac{f_{0}}{2}\right)\left(\frac{\Delta k}{k_{0}}-\frac{\Delta m}{m_{0}}\right)\approx \frac{f_{0}}{2k_{0}}\Delta k$$

where *f*_0_, *k*_0_, and *m*_0_ are the resonance frequency (32.76 kHz), spring constant (13 kN m^−1^), and mass (~2.7 mg) of the bare QTF, respectively. Note that the signs of Δ*f* and Δ*m* are opposite. As the addition of a 2 μm-thick and 200 µm-long P30 fiber increases the mass by 2 ng, the frequency of the QTF should decrease by 0.01 Hz if the spring constant is unchanged. When a polymer fiber was cut by a razor blade, the resonance frequency of the QTF with the polymer fiber residue was almost identical to that of the bare QTF, indicating that the mass effect was not significant. However, the attachment of the P30 fiber to the QTF induced an increase in the frequency [16]. Figure 3b shows that the resonance frequencies of the bare QTF (32.76 kHz) increased by 47, 90, 177, and 420 Hz, respectively, upon the coating of P60, P30, CP60, and CP30 fibers, indicating that the mass loading effect is negligible when compared with the stiffness effect. The apparent modulus of the polymer fiber can be calculated from the changes in the spring constant, as follows [8]:

(2) $$E=\frac{2Lk_{0}}{2f_{0}}\Delta f\approx \frac{L}{A}\Delta k$$

where *L* and *A* are the length and apparent cross-sectional area of the fiber, respectively. The apparent modulus was calculated to be 2.1, 4.0, 8.2, and 17.6 GPa for the P60, P30, CP60, and CP30 fibers, respectively. The electrospun fibers prepared at a high humidity are mechanically weaker than those prepared at a low humidity, because of the presence of surface pores and inner cavities.

### 3.3. Effect of CNC Contents on the Sensor Performance

**Figure 4:**
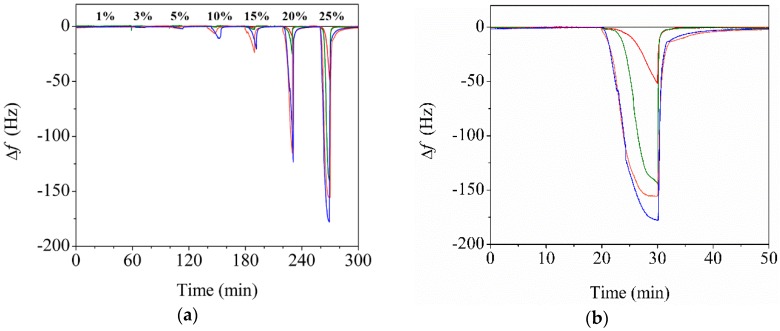
(**a**) Time-dependent changes in the resonance frequencies of QTF (black), P30-QTF (red), 0.5CP30-QTF (green), 1CP30-QTF (orange), and 3CP30-QTF (blue) upon exposure to ethanol vapor. The concentrations of ethanol vapor were varied sequentially at each of the following measurements: 1%, 3%, 5%, 10%, 15%, 20%, and 25%. (**b**) Time-dependent changes in the resonance frequency of QTFs when exposed to 25% ethanol vapor.

Figure 4a shows the changes in the resonance frequencies (i.e., changes in the modulus) of the bare QTF, P30-QTF, and CP30-QTFs with different amounts of CNCs in PMMA (0.5 wt%, 0.5CP30-QTF; 1.0 wt%, 1CP30-QTF; and 3.0 wt%, 3CP30-QTF) when exposed to ethanol vapor. Ethanol vapor was injected into the flow cell for 10 min to allow for the adsorption of ethanol vapor, and dry nitrogen was then injected for 30 min to remove the ethanol vapor. The frequency decreased during the adsorption of ethanol vapor and the frequency change increased as the ethanol vapor concentration increased. The frequencies recovered to their original values after the removal of ethanol vapor, confirming that ethanol adsorption on the electrospun fibers is reversible.

Figure 4b magnifies the frequency changes of the QTFs upon exposure to 25% ethanol vapor. Only a negligible change was observed for the bare QTF, as it responded to mass loading but its mass sensitivity (~50 ng Hz^−1)^ was too low to measure the adsorption of the ethanol vapor. In contrast, the electrospun fiber-coated QTFs responded to ethanol vapor and produced changes in the resonance frequency. This frequency change was caused not by an adsorption-induced change in mass loading, but by an adsorption-induced change in the modulus of the electrospun fiber. The frequency change increased when increasing the CNC content, which confirmed that the frequency change was caused by the modulus change of the fiber. In addition, the response time (i.e., the time required for the frequency to reach 63.2% of the maximum change) of CP30-QTF was faster than that of P30-QTF, regardless of the CNC content. The response time of 3CP30-QTF was 223 s when exposed to a 30% ethanol atmosphere, which is almost twice as fast as that of P30-QTF (465 s). The fast response time of three CP30-QTFs was attributed to the presence of pore networks that enabled the gas molecules to diffuse through the CP-30 faster than through the plain P-30.

### 3.4. Enhanced Performance of the Reinforced Nanoporous Fiber-Mount QTF

**Figure 5:**
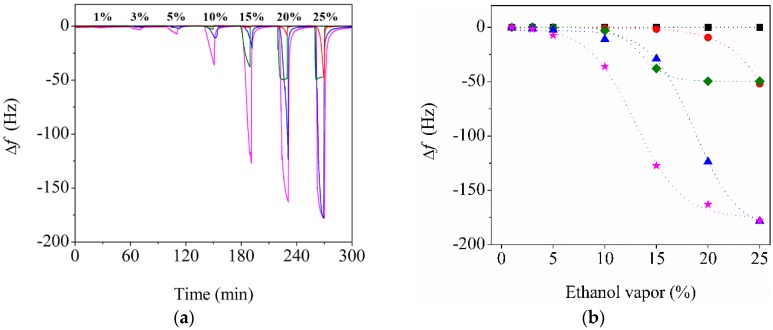
(**a**) Time-dependent changes in the resonance frequency changes of the bare QTF (black), P30-QTF (red), P60-QTF (green), CP30-QTF (blue), and CP60-QTF (pink) upon exposure to ethanol vapor. The concentrations of ethanol vapor were varied sequentially at each of the following measurements: 1%, 3%, 5%, 10%, 15%, 20%, and 25%. (**b**) Changes in the resonance frequencies of the bare QTF (black square), P30-QTF (red circle), P60-QTF (green diamond), CP30-QTF (blue triangle), and CP60-QTF (pink star) upon exposure to ethanol vapor. Dashed curves were made to guide the eye.

The porosity of the polymer fiber was substantially affected by the RH during electrospinning. Figure 5a shows the changes in the resonance frequencies of the bare QTF, P30-QTF, P60-QTF, CP30-QTF, and CP60-QTF when they were exposed to ethanol vapor with different concentrations. The frequency changes increased as the ethanol vapor concentration increased. The response time of CP60-QTF upon exposure to a 20% ethanol atmosphere was 48 s, which is more than 4.7 times faster than that of CP30-QTF (223 s). This fast response time confirms that the diffusion of gas molecules was facilitated by the presence of three-dimensionally connected pores. Figure 5b compares the frequency shifts of the five QTFs with the concentration of the ethanol vapor. With ethanol vapor concentrations below 20%, the frequency changes of the P30-QTF and CP30-QTF were smaller than those of P60-QTF and CP60-QTF, respectively. However, the frequency changes became similar at a 25% ethanol atmosphere, because of the saturation of P60-QTF and CP60-QTF with ethanol vapor. P30-QTF and CP30-QTF were not saturated at the 25% ethanol atmosphere, because of their relatively dense structure. 

### 3.5. Water Sensing Performance

**Figure 6:**
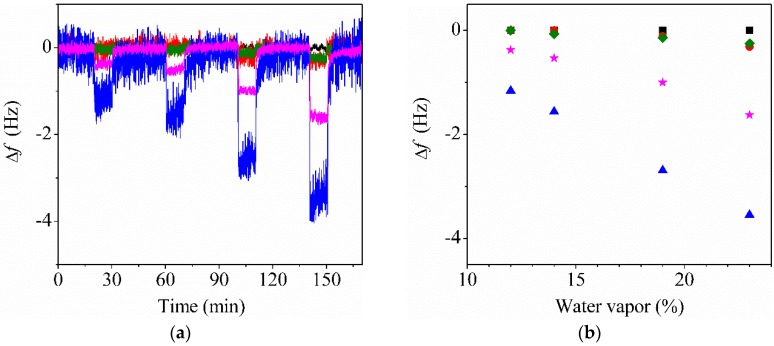
(**a**) Time-dependent changes in the resonance frequency changes of the bare QTF (black), P30-QTF (red), P60-QTF (green), CP30-QTF (blue), and CP60-QTF (pink) upon exposure to water vapor. The relative humidity (RH) was varied sequentially at each of the following measurements: 9%, 12%, 14%, 19%, and 23%. (**b**) Changes in the resonance frequencies of the bare QTF (black square), P30-QTF (red circle), P60-QTF (green diamond), CP30-QTF (blue triangle), and CP60-QTF (pink star) upon exposure to water vapor.

A control experiment was carried out to investigate the influence of moisture on the frequency changes. Figure 6 shows that the frequency changes of the bare QTF, P30-QTF, P60-QTF, CP30-QTF, and CP60-QTF were measured after exposure to different RHs. As with ethanol, negligible changes were observed for the bare QTF, regardless of the RH. Notably, the frequency changes of the fiber-coated QTFs due to moisture adsorption were considerably smaller than those due to ethanol vapor adsorption. However, the response time of the fiber-coated QTFs was faster than that with ethanol, and the frequency change was fully saturated at each concentration. The differences were attributed to the higher affinity of PMMA with ethanol than with water, indicating that water was adsorbed only on the PMMA surface and did not penetrate the PMMA.

## 4. Conclusions

We demonstrated that both the sensitivity and response time of a QTF used as a gas sensor could be improved by attaching a CNC-reinforced nanoporous polymer fiber to the QTF. It is not straightforward to improve the sensitivity and response time of a polymer fiber-coated QTF simultaneously, because the increase in the modulus of a polymer fiber enhanced the sensitivity, but resulted in a slow response time, and the increase in the surface area improved the response time but decreased the sensitivity. The problem was addressed by fabricating a CNC-reinforced nanoporous fiber that has a higher modulus and surface area than the dense electrospun fiber. The high crystallinity and modulus of the CNCs increased the modulus of the composite fiber, and the hygroscopic nature of CNCs promoted the formation of nanoporous structures. The developed sensor can be used for the quality control of the alcohol content in food during fermentation, and the strategy developed in this study has the potential for use not only for gas sensors, but also for polymer membranes for gas separation.

## Supplementary Materials

The following are available online at https://www.mdpi.com/1424-8220/20/2/437/s1.
