# Supplementary material for: Performance Enhancement of a Quartz Tuning Fork Sensor Using a Cellulose Nanocrystal-Reinforced Nanoporous Polymer Fiber
Source: Sensors (Basel). 2020 Jan 13;20(2):437. doi: 10.3390/s20020437 (PMC7014262; doi:10.3390/s20020437)
Supplement: Supplementary file 1 [file sensors-20-00437-s001.pdf]

sensors

Supplementary Material

Performance Enhancement of a Quartz Tuning Fork Sensor using a Cellulose Nanocrystal-Reinforced Nanoporous Polymer Fiber

Wuseok Kim †, Eunjin Park †, and Sangmin Jeon *

Department of Chemical Engineering, Pohang University of Science and Technology (POSTECH), 77 Cheongam-Ro, Nam-Gu, Pohang, Gyeongbuk, Republic of Korea

† The authors contributed equally to this paper.

* Correspondence: jeons@postech.ac.kr; Tel.: +82-054-279-2392

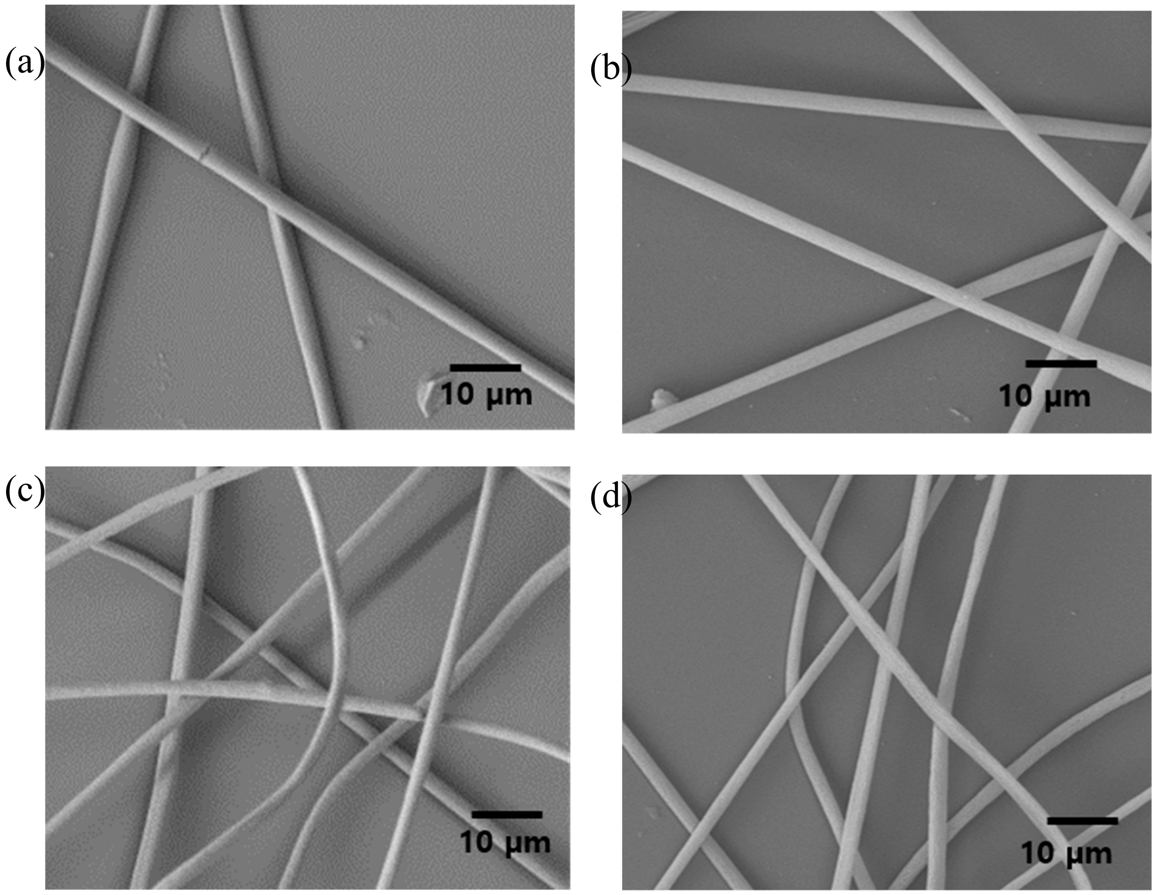

Figure S1. SEM images of (**a**) P30 fibers, (**b**) P60 fibers, (**c**) CP30 fibers, and (**d**) CP60 fibers.

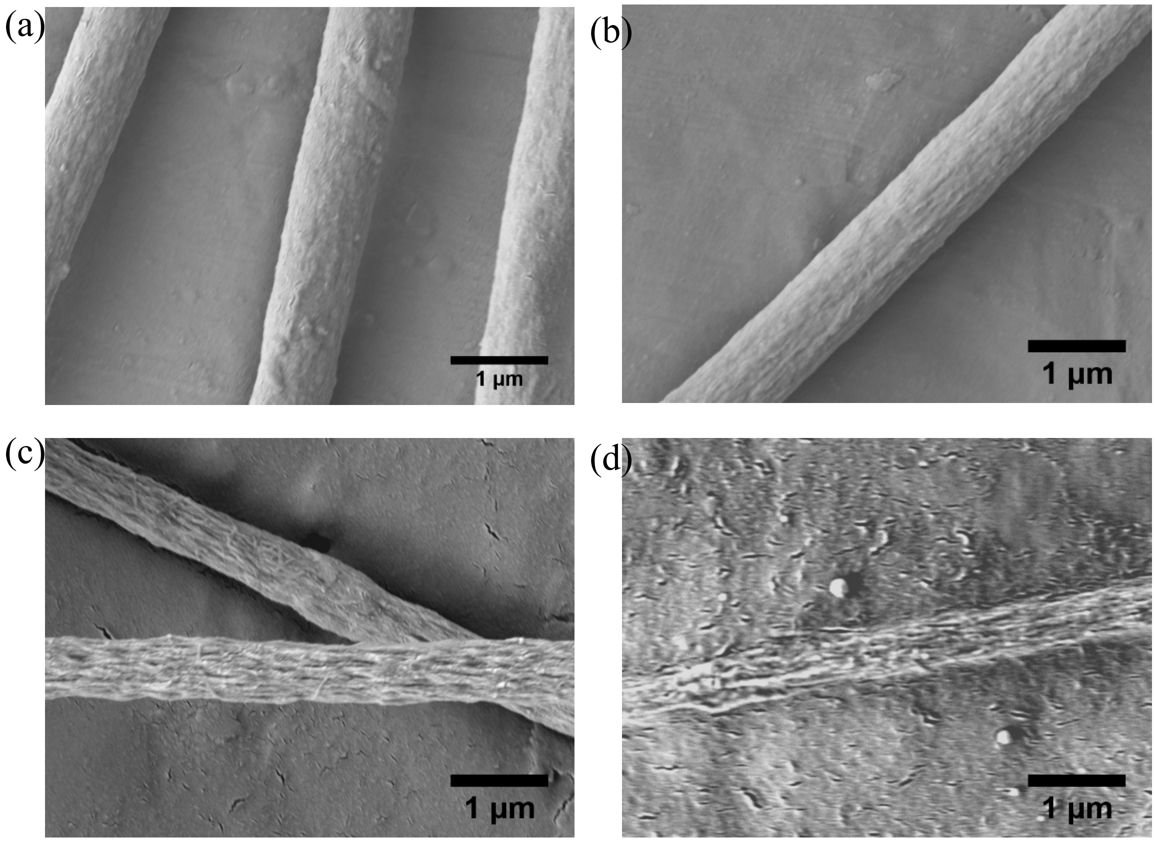

Figure S2. SEM images of CNC/PMMA fiber (3:7, *wt*/*wt*) after dropping 100 μl of THF (**a**) 1 time, (**b**) 3 times, (**c**) 5 times, and (**d**) 10 times.

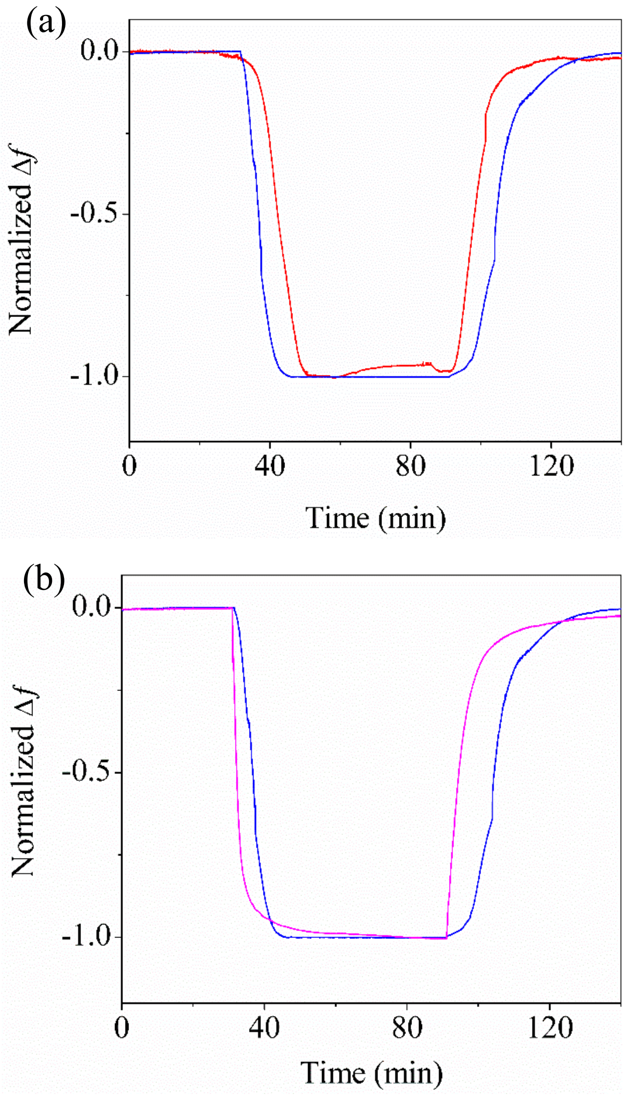

Figure S3. (**a**) Normalized frequency change of P30-QTF (red) and CP30-QTF (blue) upon exposure to 25 % ethanol vapor (**b**) Normalized frequency change of CP30-QTF (blue) and CP60-QTF (magenta) upon exposure to 25 % ethanol vapor.

Table S1. Performance of bare-, P30-, CP30-, P60-, CP60-QTFs for ethanol sensing.

	LOD	Response Time	$\|\Delta f\|$ at EtOH 25 %	SNR at EtOH 25 %
Bare QTF	20 %	-	< 0.1	7.5
P30	15 %	465 s	51.7	487.7
CP30	5 %	223 s	177.9	2869.8
P60	10 %	< 1 min	47.5	1826.9
CP60	3 %	< 1 min	173.0	2369.7
